# Machine-Learning Evaluation of a Magnetostrictive Acoustic-Emission Sensor Developed for Nuclear Structural Health Monitoring

**DOI:** 10.3390/s26185693

**Published:** 2026-09-08

**Authors:** Bibo Zhong, Chaitee Milind Godbole, Vivek Agarwal, Joshua E. Daw

**Affiliations:** Idaho National Laboratory, Idaho Falls, ID 83415, USA; bibo.zhong@inl.gov (B.Z.); chaiteemilind.godbole@inl.gov (C.M.G.); vivek.agarwal@inl.gov (V.A.)

**Keywords:** acoustic emission, impact classification, structural health monitoring, random forest, multi-sensor fusion, magnetostrictive sensor, nuclear monitoring, machine learning, feature extraction

## Abstract

Acoustic emission (AE) sensing is widely used for structural health monitoring, but conventional piezoelectric sensors can be constrained in high-temperature and radiation environments. This study evaluates whether an Idaho National Laboratory-developed magnetostrictive AE sensor retains sufficient information for automated impact-source classification under a controlled room-temperature configuration. A total of 334 synchronized acquisitions from six impact classes were recorded at 20 MHz using three fixed, non-coincident sensor channels. Because sensor type was not independently varied from position, mounting, coupling, bandwidth, or propagation path, comparisons represent complete sensor-channel responses rather than isolated transduction mechanisms. Each waveform was represented by 16 time-domain, spectral, and band-power features. Random forest classifiers were evaluated for individual channels, 48-feature fusion, and equal-vote decision fusion using 30 repeated stratified holdout runs and stratified 10-fold cross-validation. The magnetostrictive channel achieved 95.35 ± 2.45% mean holdout accuracy, compared with 92.37 ± 2.94% and 96.31 ± 1.98% for the two commercial channels. Feature-level fusion achieved 97.12 ± 2.19% and 97.91 ± 3.18% under holdout and 10-fold cross-validation, respectively. Because class-specific blocks were split at the event level, these accuracies are within-campaign and within-specimen, may be optimistic, and do not establish block-independent or operational generalization; validation using randomized or interleaved repeated blocks, multiple specimens, position-controlled comparisons with swapped, rotated, or co-located sensor placements, and harsh-environment testing remains necessary.

## 1. Introduction

Structural health monitoring (SHM) aims to assess the integrity of engineering structures by acquiring sensor data and extracting indicators of damage or changing condition. Among the available sensing modalities, acoustic emission (AE) is particularly attractive because it enables passive, real-time detection of transient events such as crack initiation, delamination, fiber breakage, and impacts without interrupting operation [1,2,3,4]. AE signals are elastic waves generated by rapid, localized stress changes and recorded by transducers attached to the monitored structure. AE monitoring has also been investigated for nuclear structures, where continuous access for conventional inspection may be limited [5].

A fundamental challenge in AE-based SHM is distinguishing among different source mechanisms from the recorded waveforms. Impactors with different masses, contact geometries, and materials can excite different elastic-wave modes and produce characteristic time- and frequency-domain responses [6,7]. Manual interpretation of large AE datasets is time-consuming and may be influenced by operator judgment, motivating the development of automated, data-driven classification methods [8,9].

Conventional AE systems commonly use piezoelectric transducers. However, piezoelectric sensing materials can experience degradation under neutron and gamma irradiation [10]. Idaho National Laboratory (INL) has developed a Fe-Co-alloy magnetostrictive AE sensor that operates through the inverse magnetostrictive, or Villari, effect [11,12]. Previous work demonstrated fuel-pin burst detection at temperatures up to 650 °C [11], while irradiation studies of related magnetostrictive materials support the harsh-environment potential of magnetostrictive sensing materials [10]. The present study does not independently test elevated-temperature or irradiation performance and therefore does not establish that classification accuracy is retained under those conditions. The narrower question addressed here is whether the INL magnetostrictive sensor channel retains sufficient discriminative information for automated source classification under a controlled room-temperature laboratory configuration.

Machine-learning methods are increasingly used to automate AE interpretation. Support vector machines and random forest classifiers have been applied to frequency-based AE classification and damage-mode identification [13,14,15,16]. Convolutional neural networks have also been investigated using image-transformed representations such as wavelet scalograms and PCA-derived maps [17,18]. Because the present dataset contains a relatively limited number of synchronized laboratory events, this study uses a compact set of physically motivated features with random forest classification, providing a computationally efficient and interpretable alternative to more data-intensive image-based models.

Multi-sensor measurements can further improve classification by combining information from channels with different bandwidths, sensitivities, propagation paths, and coupling characteristics. Data-level fusion of multiple AE sensor signals has been investigated using deep-learning-based reconstruction methods [18], and heterogeneous ensemble learning has been applied to other AE damage-assessment problems [19]. However, to the authors’ knowledge, synchronized magnetostrictive and piezoelectric sensor channels have not previously been evaluated together for AE impact-source classification. The present experiment therefore compares and fuses three simultaneously acquired sensor channels under a common room-temperature configuration. Because the sensors occupied fixed, non-coincident positions, the study evaluates the complete channel responses, including the combined effects of transduction mechanism, bandwidth, sensor position, mounting, coupling, and propagation path. Accordingly, the methodological contribution is not a new classifier or fusion algorithm; it is an evaluation of the information retained by this magnetostrictive AE channel and its behavior within a synchronized multi-sensor classification framework.

The principal contributions of this work are as follows:

- A systematic machine-learning evaluation of an INL-developed magnetostrictive AE channel alongside commercial high-temperature and broadband piezoelectric channels for six-class impact-source classification under one synchronized room-temperature configuration;
- Within-campaign comparison of individual sensor models, 48-feature sensor fusion, and equal-vote decision fusion using 30 repeated stratified holdout runs and stratified 10-fold cross-validation, with feature-level fusion achieving mean accuracies of 97.12 ± 2.19% and 97.91 ± 3.18%, respectively;
- Predictor-count and feature-redundancy sensitivity analyses showing near-plateau performance with approximately 12–16 training-ranked fused predictors and negligible accuracy change after removing the three energy predictors.

The remainder of this paper is organized as follows. Section 2 reviews related work. Section 3 describes the experimental configuration and dataset. Section 4 presents the feature-extraction methodology. Section 5 describes the classification models, fusion strategies, and evaluation protocol. Section 6 reports the results, Section 7 discusses their interpretation and limitations, and Section 8 summarizes the principal conclusions and future work.

## 2. Related Work

### 2.1. Feature Extraction for Acoustic Emission Signals

AE features are commonly grouped into time- and frequency-domain descriptors. Time-domain parameters—including amplitude, root-mean-square value, energy, rise time, duration, threshold counts, kurtosis, and skewness—are widely used because they can be computed efficiently and relate to waveform amplitude, duration, and impulsiveness [20]. Frequency-domain descriptors, such as peak frequency, mean frequency, spectral centroid, and bandwidth, are obtained from the Fourier spectrum and characterize differences in modal and spectral content [6,20]. Band-power features further partition the spectrum into predefined frequency intervals to capture changes associated with source mechanism and wave propagation [21].

More complex representations have also been investigated, including wavelet-transform features [7], wavelet-packet features [22], empirical-mode-decomposition features [9], and short-time Fourier transform spectrograms [8]. These approaches can describe non-stationary AE behavior in greater detail but generally increase representation size and computational complexity. Unsupervised pattern-recognition methods, including natural-cluster identification and k-means-based damage characterization, have also been applied directly to AE feature spaces when labeled observations are limited [23,24]. For the relatively small dataset and the emphasis on physically interpretable processing in the present study, a compact set of time- and frequency-domain features was selected.

### 2.2. Machine-Learning Classifiers for AE Impact Identification

Support vector machines with radial-basis-function kernels have been applied to frequency-based AE classification [15]. Random forests have also been used for AE damage classification and clustering [16]. Their intrinsic multiclass capability, limited sensitivity to feature scaling, and out-of-bag importance measures make them suitable for compact engineered-feature datasets [14].

Convolutional neural networks have been investigated using image-transformed AE representations, including continuous-wavelet-transform scalograms and PCA-derived maps [17,18]. Such approaches can reduce reliance on handcrafted scalar features but generally require larger datasets and more computational resources than conventional classifiers. Recent studies have also applied supervised machine-learning to multilayer damage detection [25] and fatigue-stage characterization [26]. Embedded and TinyML implementations have been explored to reduce the computational burden of on-site AE monitoring [27]. Given the size of the present dataset and the need for an interpretable, computationally efficient classifier, random forest classification was adopted in this study.

### 2.3. Multi-Sensor Fusion for AE Classification

AE localization has been investigated using deep-learning methods, including approaches based on a single sensor [28]. For classification and signal reconstruction, data-level fusion of multiple AE sensor signals has been explored using deep learning [18]. Heterogeneous ensemble learning has also been applied to AE-based concrete damage assessment, although that work combined multiple classifiers rather than sensor channels operating through different transduction mechanisms [19].

These studies demonstrate the potential value of integrating complementary AE information. However, to the authors’ knowledge, synchronized piezoelectric and magnetostrictive sensor channels have not previously been evaluated together for impact-source classification. The present study addresses this gap by comparing the individual channels and evaluating feature-level and decision-level fusion under a common acquisition configuration. Because each sensor occupied a fixed, non-coincident location, the resulting comparison represents the complete channel response, including sensor bandwidth, transduction mechanism, position, propagation path, mounting, and coupling; it is not a position-controlled comparison of transduction mechanisms.

### 2.4. Magnetostrictive Sensing for AE

Magnetostrictive transducers convert mechanical strain into changes in magnetic flux through the inverse magnetostrictive, or Villari, effect. Like contact piezoelectric sensors, they require mechanical coupling to the monitored structure; however, their metallic sensing elements can offer advantages in high-temperature and radiation environments.

Previous INL work demonstrated passive fuel-pin burst detection using a Fe-Co-alloy magnetostrictive AE sensor at temperatures up to 650 °C [11]. Related design development also demonstrated active pulse-echo ultrasonic operation at higher temperatures and introduced ceramic-coated coil wires, acoustic-coupling amplifiers, active self-testing, and bias-coil sensitivity enhancement [12]. Irradiation testing of related magnetostrictive materials has shown tolerance to neutron and gamma exposure [10]. The approximately 49% Fe–49% Co–2% V alloy used in the sensor has a Curie temperature near 950 °C [11,12], while the commercial high-temperature piezoelectric sensor used for comparison in this study is rated to 540 °C [29].

These prior investigations establish the harsh-environment potential of the magnetostrictive sensor architecture. They do not, however, determine whether its measured AE signals retain sufficient information for automated source classification. To the authors’ knowledge, the present work provides the first systematic machine-learning evaluation of this INL magnetostrictive sensor channel alongside commercial piezoelectric alternatives using a synchronized, six-class impact dataset. The present experiments are limited to room temperature and are not a validation of classification performance under irradiation or elevated temperature.

## 3. Experimental Setup and Data Acquisition

### 3.1. Test Structure and Sensors

The experimental test structure was a 304 stainless-steel block measuring 203.2 × 203.2 × 25.4 mm (8 × 8 × 1 in). Three AE sensors were mounted simultaneously on the block so that each source event was recorded synchronously by all three channels. The D9215 and WD commercial piezoelectric AE sensors were obtained from MISTRAS Group, Inc. (Princeton Junction, NJ, USA), while the magnetostrictive AE sensor was developed in-house at Idaho National Laboratory (INL; Idaho Falls, ID, USA). The sensor types and key specifications are summarized in Table 1.

The frequency ranges listed in Table 1 are manufacturer-reported response or operating ranges, not calibrated transfer functions for the complete mounted acquisition channels. The WD datasheet distinguishes a 100–900 kHz good-response range from a 125–1000 kHz operating range. No sensor-specific frequency masking was applied; the same 0–700 kHz digital bins were retained for all channels after the common 30–700 kHz analog conditioning, including frequency portions outside the manufacturer-reported response or operating ranges. No independent transfer-function measurement of the complete sensor–mounting–coupling–preamplifier chains was performed; spectral comparisons are therefore interpreted as empirical complete-channel responses under the common conditioning used in this study.

The three sensors were mounted at fixed, non-coincident locations. Consequently, the comparisons reported in this study represent the complete sensor-channel responses under the tested configuration, including the combined effects of transduction mechanism, sensor bandwidth, spatial position, propagation path, mounting, and coupling. The experiment was not designed to isolate transduction mechanism as the sole cause of differences among the recorded sensor responses, and the reported channel-to-channel accuracy differences should not be interpreted as intrinsic transducer-performance differences.

All three channels were digitized synchronously at 20 MHz, corresponding to a Nyquist frequency of 10 MHz. This sampling frequency is well above the 700 kHz upper frequency retained for the subsequent feature analysis.

### 3.2. Impact Classes

Six repeatable laboratory source classes were applied to the stainless-steel block. The classes were selected to produce different contact geometries, energy levels, and waveform characteristics:

- Big steel ball (BSB): 10 mm-diameter low-carbon steel sphere, producing relatively high-amplitude, broadband, and long-duration AE signals;
- Small steel ball (SSB): 3 mm-diameter low-carbon steel sphere, producing lower-energy and shorter-duration signals than the BSB source;
- Metal wire (Wire): 0.6 mm diameter tungsten wire contact; produces a distinctive high-frequency signature;
- Pencil 0.3 mm (P3): 0.3 mm pencil-lead break, producing a sharp, localized AE source;
- Pencil 0.5 mm (P5): 0.5 mm pencil-lead break, producing an intermediate-energy pencil source;
- Pencil 0.7 mm (P7): 0.7 mm pencil-lead break, producing the highest-energy pencil-lead source among the three pencil classes.

These controlled sources served as repeatable laboratory surrogates for evaluating source-classification performance. They were not intended to reproduce specific reactor damage mechanisms directly.

### 3.3. Data Acquisition

Signals from all three sensors were amplified using MISTRAS 2/4/6 preamplifiers (MISTRAS Group, Inc., Princeton Junction, NJ, USA) with a gain of 60 dB and a 30–700 kHz bandpass filter. The amplified signals were digitized synchronously on all three channels using a PicoScope 4824A oscilloscope (Pico Technology Ltd., St Neots, UK). Each acquisition contained 1,100,000 samples per channel at a sampling frequency of 20 MHz, corresponding to a total recording duration of 55 ms. The common analog conditioning therefore strongly attenuated content below approximately 30 kHz and above 700 kHz for all three channels.

Acquisition was triggered when any sensor channel exceeded a threshold of 0.05 V. Each record included a 5 ms pre-trigger interval and a 50 ms post-trigger interval. Because the three channels shared the same trigger and temporal reference, every impact event produced one synchronized three-channel acquisition. The acquisition terminology follows ASTM E1316-21 [31].

Data were collected at room temperature, and the six impact-source classes were acquired in class-specific blocks rather than in randomized or interleaved order. Approximately 57 events were targeted per class. After data-quality screening, 334 synchronized acquisitions were retained, with approximately 55–57 usable events per class. Because the campaign did not include independently randomized or interleaved repeated blocks for each class, class identity cannot be separated retrospectively from possible between-block changes in coupling, instrumentation, or other experimental conditions. Feature extraction was performed on the complete 55 ms waveform after mean removal. The acquisition parameters are summarized in Table 2.

**Figure 1:**
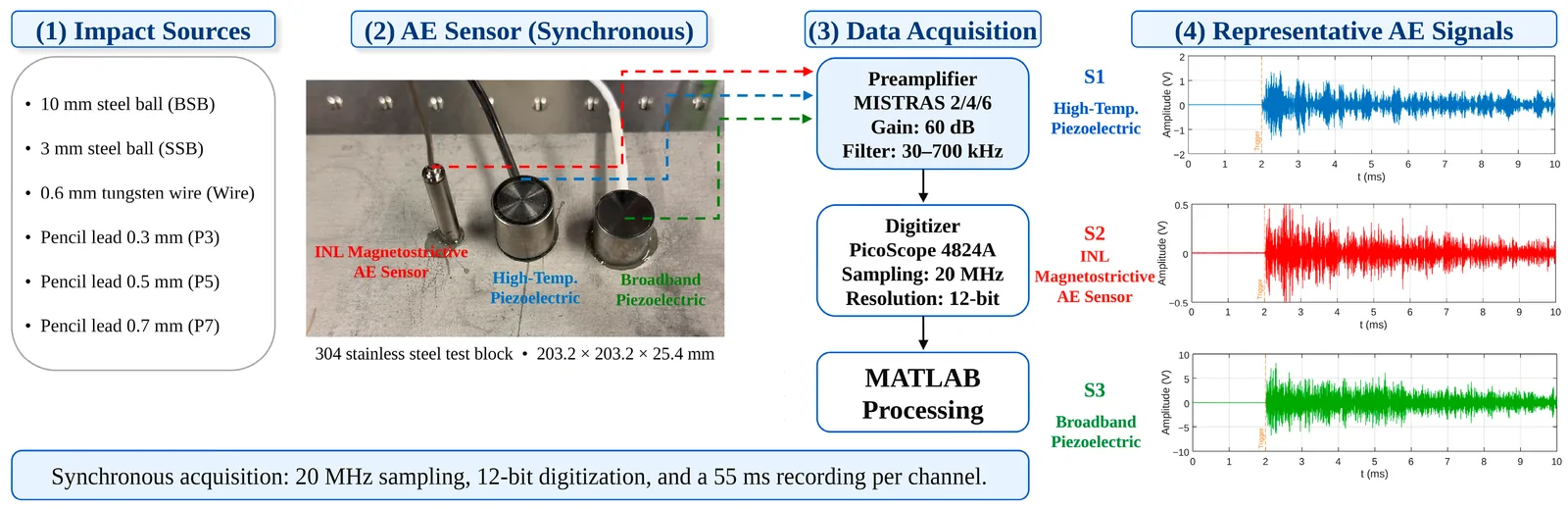
Experimental setup and representative synchronized waveforms for a small-steel-ball impact.

Figure 1 summarizes the experimental configuration, including the six laboratory source classes, the three simultaneously mounted AE sensors, the data acquisition hardware, and representative synchronized waveforms from a small-steel-ball event.

## 4. Feature Extraction Methodology

A total of 16 features were extracted independently from each sensor waveform. The feature set comprised seven time-domain descriptors, four general frequency-domain descriptors, and five band-power features. Feature extraction was performed on the complete 55 ms record, containing 1,100,000 samples per channel, after removal of the waveform mean.

Because the signals were conditioned using a 30–700 kHz analog bandpass filter, only spectral content at or below 700 kHz was retained for frequency-domain feature extraction. The lowest nominal integration interval was defined as 0–100 kHz, but content below approximately 30 kHz was strongly attenuated by the common analog high-pass filter; this feature therefore primarily represents the approximately 30–100 kHz portion of the acquired spectrum. Manufacturer-reported sensor frequency ranges were treated as nominal response or operating ranges rather than brick-wall cutoffs. No sensor-specific frequency masking was applied; the same 0–700 kHz digital bins were retained for all channels after the common 30–700 kHz analog conditioning, including frequency portions outside the manufacturer-reported response or operating ranges. Because complete-channel transfer functions were not measured, the spectral features are interpreted as empirical responses of the mounted sensor–coupling–preamplifier channels rather than calibrated sensor sensitivity functions. The resulting feature vector for each waveform was defined as:

$$f={\left[f_{1},f_{2},\ldots ,f_{16}\right]}^{T}\;,$$

where $f_{i}$ denotes the *i*-th extracted feature.

All data processing and feature extraction were performed using MATLAB R2024b (The MathWorks, Inc., Natick, MA, USA). A small positive constant, *ϵ*, was added to selected denominators to prevent division by zero. ϵ corresponds to MATLAB’s built-in double-precision machine epsilon ($\approx 2.22\times 10^{-16}$).

### 4.1. Time-Domain Features ($f_{1}–f_{7}$)

Let $x\left[n\right],\;n\;=\;1,\ldots ,N$, denote the discrete AE signal of length N = 1,100,000 samples after mean removal.

The seven time-domain features are:

**RMS (** $f_{1}$ **)**

The root-mean-square (RMS) value represents the effective amplitude of the waveform.

$$f_{1}=\sqrt{\frac{1}{N}\sum_{n=1}^{N}x^{2}\left[n\right]}$$

**Peak amplitude (** $f_{2}$ **)**

Peak amplitude is defined as the maximum absolute waveform amplitude.

$$f_{2}=\max\left(\left|x\left[n\right]\right|\right)$$

**Signal energy (** $f_{3}$ **)**

Signal energy represents the cumulative waveform energy over the acquisition window.

$$f_{3}=\sum_{n=1}^{N}x^{2}[n]\;$$

**Crest factor (** $f_{4}$ **)**

The crest factor measures waveform impulsiveness by comparing the peak amplitude with its RMS value.

$$f_{4}=\frac{f_{2}}{f_{1}+\epsilon }$$

**Kurtosis (** $f_{5}$ **)**

Kurtosis is the fourth standardized statistical moment and is particularly sensitive to transient impulsive events.

$$f_{5}=\frac{\frac{1}{N}\sum_{n=1}^{N}{\left(x\left[n\right]-\mu \right)}^{4}}{{\left(\sigma \right)}^{4}}$$

where:

$$\mu =\frac{1}{N}\sum_{n=1}^{N}x\left[n\right]$$

and:

$$\sigma =\sqrt{\frac{1}{N-1}\sum_{n=1}^{N}{\left(x\left[n\right]-\mu \right)}^{2}}$$

**Normalized Peak Time (** $f_{6}$ **)**

Normalized peak time describes the temporal position of the maximum absolute waveform amplitude within the 55 ms acquisition:

$$f_{6}=\frac{n_{peak}}{N}\;,$$

where $n_{peak}$ is the sample index corresponding to the maximum absolute waveform amplitude. Because all three channels shared a common acquisition trigger, this feature represents timing relative to the same temporal reference for every sensor channel.

**Normalized active duration (** $f_{7}$ **)**

The normalized active duration is defined as the fraction of waveform samples whose absolute amplitude exceeds 10% of the peak amplitude:

$$f_{7}=\frac{1}{N}\sum_{n=1}^{N}I(\left|x\left[n\right]\right|>0.1f_{2})\;,$$

where I(⋅) is the indicator function.

Because all records had the same length and both RMS and energy were calculated over the same analysis window, the two features are mathematically related:

$$f_{3}=Nf_{1}^{2}.$$

Both features were retained in the full feature set for consistency with the original analysis; however, they should not be interpreted as independent physical descriptors.

### 4.2. Frequency-Domain Features ($f_{8}–f_{16}$)

The one-sided amplitude spectrum was computed using the discrete Fourier transform:

$$X\left[k\right]\;=\left|\sum_{n=0}^{N-1}x\left[n\right]e^{-\frac{j2\pi kn}{N}}\right|,$$

with corresponding frequency bins:

$$f_{k}=\frac{kf_{s}}{N}\;,$$

where $f_{s}$ is the sampling frequency.

Only frequencies satisfying:

$$0\leq f_{k}\leq 700\;kHz$$

were retained for feature extraction. Let K denote the number of retained one-sided frequency bins.

**Mean frequency (** $f_{8}$ **)**

Mean frequency is the amplitude-weighted average frequency of the retained spectrum.

$$f_{8}=\frac{\sum_{k=0}^{K-1}f_{k}X[k]}{\sum_{k=0}^{K-1}X[k]+\epsilon }\;$$

**Spectral centroid (** $f_{9}$ **)**

Spectral centroid is the power-weighted center of the retained spectrum.

$$f_{9}=\frac{\sum_{k=0}^{K-1}f_{k}X^{2}\left[k\right]}{\sum_{k=0}^{K-1}X^{2}\left[k\right]+\epsilon }\;,$$

**Spectral bandwidth (** $f_{10}$ **)**

Spectral bandwidth measures the spread of spectral energy around the spectral centroid.

$$f_{10}=\sqrt{\frac{\sum_{k=0}^{K-1}{\left(f_{k}-f_{9}\right)}^{2}X^{2}\left[k\right]}{\sum_{k=0}^{K-1}X^{2}\left[k\right]+\epsilon }}\;,$$

**Band-power ratio (** $f_{11}$ **)**

The band-power ratio quantifies the balance between the primary AE extensional-wave band (100–300 kHz) and higher-frequency components (300–700 kHz):

$$f_{11}=\frac{P_{100-300}}{P_{300-700}+\epsilon }\;,$$

where:

$$P_{a-b}=\sum_{f_{k}\in [a,b]}X^{2}\left[k\right].$$

**Band-Power Features (** $f_{12}$ **–** $f_{16}$ **)**

The remaining five spectral features correspond to energy contained within predefined frequency bands:

$$f_{i}=\sum_{f_{k}\in B_{i}}X^{2}\left[k\right],\;\;\;\;i=12,\ldots ,16,$$

where:

$$B_{i}\in \left\{\left[0,100),[100,200),[200,300),[300,500),[500,700\right)\right\}\;kHz,$$

as summarized in Table 3.

### 4.3. Feature Normalization

Prior to model training, each feature was standardized using z-score normalization:

$$z_{i}=\frac{f_{i}-\mu_{i}}{\sigma_{i}+\epsilon }\;,$$

where $\mu_{i}$ and $\sigma_{i}$ denote the mean and standard deviation of the i-th feature computed exclusively from the training partition.

The normalization parameters ($\mu_{i},\sigma_{i}$) were stored together with the trained model and applied unchanged during inference to ensure identical preprocessing for unseen data. For feature-level fusion, the 16 features from each sensor channel were standardized separately using that channel’s training-partition statistics before the three normalized vectors were concatenated into the 48-dimensional fused representation.

## 5. Classification Models and Fusion Strategies

Three random forest configurations were evaluated: individual sensor-channel models, feature-level fusion, and decision-level fusion. For each synchronized event, the feature-extraction procedure described in Section 4 produced one standardized 16-dimensional feature vector per sensor channel.

### 5.1. Single-Sensor Random Forest Baseline

A random forest (RF) classifier [14] was trained independently for each of the three sensor channels. Each model used the corresponding channel’s 16 standardized predictors to classify the event into one of the six impact-source classes.

Each RF contained 100 decision trees. Every tree was trained using a bootstrap sample of the training observations, and the number of candidate predictors considered at each split was defined using the standard square-root rule:

$$m_{try}=\left\lfloor \sqrt{p}\right\rfloor ,$$

where p denotes the number of input predictors. For the individual sensor models, p=16.

The predicted class was determined by majority vote across the trees. Out-of-bag (OOB) error was monitored as a function of tree count during model development. Because the OOB error stabilized before 100 trees, 100 trees were used for all subsequent models.

Independent baseline models were trained for:

- S1, the high-temperature commercial piezoelectric channel;
- S2, the INL magnetostrictive channel; and
- S3, the broadband commercial piezoelectric channel.

### 5.2. Feature-Level Fusion (FF-RF)

For feature-level fusion, the three independently standardized 16-dimensional sensor-channel vectors were concatenated to form a single 48-dimensional representation:

$$z_{FF}={\left[\begin{array}{ccc} z_{S1}^{T} & z_{S2}^{T} & z_{S3}^{T} \end{array}\right]}^{T}\in R^{48}.$$

A single RF classifier was then trained using the fused vector. The same 100-tree configuration and square-root predictor-selection rule used for the individual sensor models were applied to the feature fusion model, with p=48.

Feature-level fusion allows the classifier to learn relationships among predictors from different sensor channels, including channel-specific amplitude, temporal, and spectral responses.

### 5.3. Decision-Level Fusion

Decision-level fusion retained three independently trained RF models, one for each sensor channel. For an event j, the three models produced predictions:

$${\hat{y}}_{j}^{\left(S1\right)},{\hat{y}}_{j}^{\left(S2\right)},{\hat{y}}_{j}^{\left(S3\right)}.$$

The fused prediction was the class receiving the largest number of votes:

$${\hat{y}}_{j}^{\left(DF\right)}=arg\;maxc\sum_{s\in \{S1,S2,S3\}}I\left({\hat{y}}_{j}^{\left(s\right)}=c\right),$$

where c denotes one of the six source classes and I(⋅) is the indicator function.

When all three models predicted different classes, the implementation used the alphabetically first class as a deterministic tie-breaking rule. No three-way voting ties occurred during the reported evaluations.

### 5.4. Training and Evaluation Protocol

Model performance was assessed using two complementary stratified validation procedures.

**Repeated stratified holdout**

A stratified 80/20 train–test split was repeated 30 times using different random seeds. Stratification approximately preserved the class distribution in every partition. For each run, the same training and test observations were used for all individual-channel and fusion models, enabling split-matched performance comparisons.

Within each run:

1. The training and test indices were generated;
2. Feature-normalization parameters were calculated using only the training observations;
3. Those parameters were applied unchanged to the corresponding test observations;
4. The RF models were trained using the training partition; and
5. Classification accuracy was calculated using the held-out test partition.

Classification accuracy was defined as:

$$Accuracy=\frac{N_{correct}}{N_{test}}\times 100\%,$$

where $N_{correct}$ is the number of correctly classified test observations and $N_{test}$ is the total number of test observations.

The mean and standard deviation of the 30 test accuracies were reported for each model. The repeated-holdout predictions were also used for a descriptive decision-fusion diagnostic. For each run, we counted (i) test events for which at least one individual-channel model was incorrect, (ii) the subset of those events classified correctly by equal-vote fusion, and (iii) fusion errors that occurred despite at least one individual-channel model predicting the true class. These quantities were summarized as mean ± standard deviation across runs and were not treated as independent inferential observations. For each run, the correction percentage was calculated as 100 times the number of events in category (ii) divided by the number in category (i); the reported percentage is the mean ± standard deviation of the 30 run-wise percentages.

**Stratified 10-fold cross-validation**

The complete dataset was also partitioned into 10 stratified folds. Each fold served once as the held-out test set, while the remaining nine folds were used for training. Feature normalization was recalculated using only the nine training folds in each iteration and then applied to the holdout fold. The same fold assignments were used for all evaluated models.

Mean accuracy and standard deviation were calculated across the 10 held-out folds. Agreement between repeated holdout and 10-fold cross-validation was interpreted as evidence of internal consistency within the present laboratory dataset, rather than evidence of external generalizability to other structures or operating environments.

Because the six source classes were acquired in class-specific blocks, random holdout and stratified cross-validation can place events from the same acquisition block in both training and test partitions. Because events rather than independently acquired blocks were partitioned, these procedures measure only internal, within-campaign, and within-specimen classification consistency. The resulting event-level holdout and cross-validation accuracies may be optimistic and do not establish block-independent, independent-session, or operational generalization. A valid group-wise assessment of acquisition-session effects would require multiple independently acquired blocks per class, preferably randomized or interleaved, which were not part of the present campaign. This design limitation cannot be removed statistically after acquisition and is discussed further in Section 7.2. Because the original signal files were retained without intentional reordering, row order within each class-specific file was used as a proxy for acquisition sequence within that block; this proxy was not independently verified against acquisition timestamps or a separate run log. Spearman’s rank correlation coefficient (ρ) was calculated between the within-file row-order proxy and each extracted feature separately for each impact class and sensor channel. Absolute correlation magnitudes were summarized across the 6 × 3 × 16 class–sensor–feature combinations. This diagnostic can reveal monotonic associations with the row-order proxy but cannot estimate constant between-block offsets or remove the confounding between class identity and acquisition block.

### 5.5. Predictor-Count and Feature-Redundancy Evaluation Procedure

Redundancy within the 48-dimensional fused representation and sensitivity to predictor count were evaluated using OOB permutation importance. For each repeated holdout run, predictor importance was estimated using only the training partition. The training-derived ranking was then used to select progressively larger predictor subsets:

N∈{1, 4, 8, 12, 16, 24, 32, 48}.

For each reduced predictor count, N<48, a new RF model was trained using only the predictors selected from the training-partition OOB permutation-importance ranking and was evaluated on the corresponding held-out partition. The N=48 endpoint was taken from the stored full feature fusion repeated-holdout results reported in Table 4 and served as the full-feature reference, rather than being generated through a duplicate stochastic retraining of the same 48-predictor configuration. The importance ranking was recomputed independently within every training partition so that observations in the held-out partition did not influence reduced-feature selection.

Mean test accuracy and standard deviation across the 30 repeated holdout runs were reported as a function of *N*. Because the importance ranking was recomputed within every training split, this analysis evaluates sensitivity to predictor count rather than defining one fixed compact feature set. It therefore should not be interpreted as independent validation of a specific reduced-dimensional model. Predictor-selection stability was also summarized as the frequency with which each predictor appeared among the top 16 training-derived rankings across the 30 partitions. In addition, because RMS and signal energy are deterministically related for equal-length records, a matched sensitivity analysis retrained the feature fusion model after removing the three energy predictors (one per sensor channel; 45 predictors total) using the same 30 holdout partitions. This redundancy check assessed whether retaining energy materially affected classification accuracy.

### 5.6. Exploratory Statistical Analysis Methods

For each model comparison, split-matched accuracy differences were calculated from the 30 repeated holdout runs:

$$d_{r}=a_{r}^{\left(A\right)}-a_{r}^{\left(B\right)},r=1,\ldots ,30,$$

where $a_{r}^{\left(A\right)}$ and $a_{r}^{\left(B\right)}$ denote the accuracies of models *A* and *B* on the same holdout split.

The paired differences were evaluated using:

- a two-sided paired *t*-test; and
- a two-sided Wilcoxon signed-rank test.

Because repeated holdout partitions overlap, the 30 paired observations are not fully independent. The resulting tests were therefore treated only as secondary exploratory sensitivity analyses rather than confirmatory statistical inference. Primary interpretation is based on the accuracy distributions, mean differences, and effect-size magnitudes.

Holm correction was applied separately to the six reported *t*-test *p*-values and six reported Wilcoxon *p*-values. Both raw and Holm-adjusted *p*-values are reported in Table 5 for transparency. Because the repeated splits are not independent, these *p*-values are not used as stand-alone confirmatory evidence; adjusted values below 0.05 are described only within the exploratory analysis.

Cohen’s paired-sample effect size was calculated as

$$d_{z}=\frac{\bar{d}}{s_{d}},$$

where $\bar{d}$ and $s_{d}$ are the mean and standard deviation, respectively, of the 30 split-matched accuracy differences. The sign of $d_{z}$ indicates the direction of the difference according to the ordering of the two models in each comparison. Effect-size magnitudes in Table 5 were described using |d_z_| < 0.20 as negligible, 0.20 ≤ |d_z_| < 0.50 as small, 0.50 ≤ |d_z_| < 0.80 as medium, and |d_z_| ≥ 0.80 as large.

All model training, prediction, feature-selection, and statistical analyses were performed in MATLAB R2024b.

**Figure 2:**
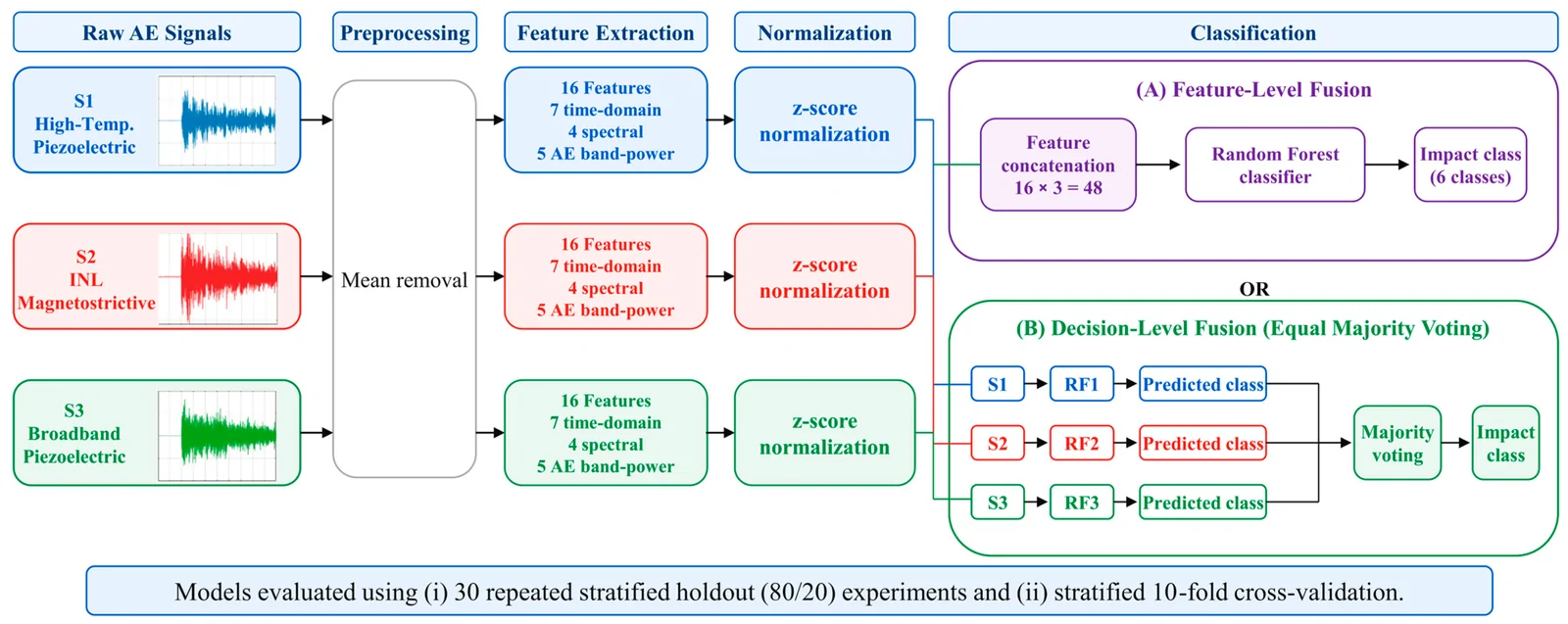
Machine-learning workflow for feature extraction, sensor-channel classification, fusion, and validation.

The complete analysis workflow is summarized in Figure 2.

## 6. Results

### 6.1. Individual-Channel and Fusion Performance

All three individual sensor-channel models achieved high classification accuracy under both validation schemes. Under repeated holdout evaluation, the high-temperature commercial channel (S1), INL magnetostrictive channel (S2), and broadband commercial channel (S3) achieved mean accuracies of 92.37 ± 2.94%, 95.35 ± 2.45%, and 96.31 ± 1.98%, respectively. The corresponding stratified 10-fold cross-validation accuracies were 93.41 ± 4.23%, 97.01 ± 3.14%, and 97.01 ± 2.84%.

The magnetostrictive-channel model therefore showed a mean accuracy 2.98 percentage points higher than that of the high-temperature commercial channel under repeated holdout evaluation and close to that of the broadband commercial channel. These numerical differences describe complete sensor-channel performance under the fixed mounting configuration and should not be attributed solely to transduction mechanism. The similar ranking under repeated holdout and 10-fold cross-validation supports only internal consistency within this single-specimen, single-campaign dataset; because events from the same class-specific block can enter both training and test partitions, these event-level accuracies may be optimistic and do not establish block-independent or operational generalization.

**Figure 3:**
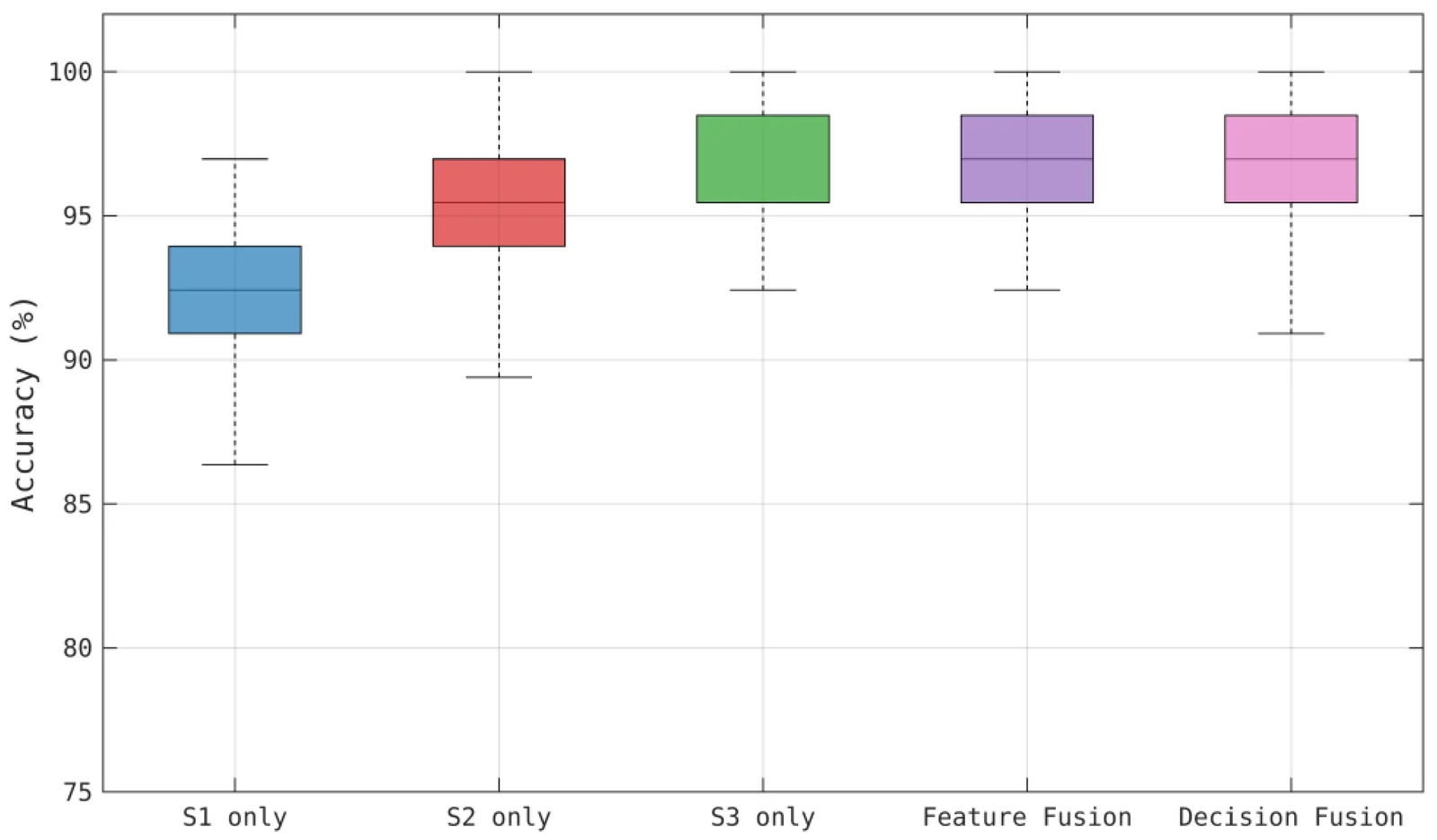
Classification accuracy across 30 repeated stratified holdout runs.

**Figure 4:**
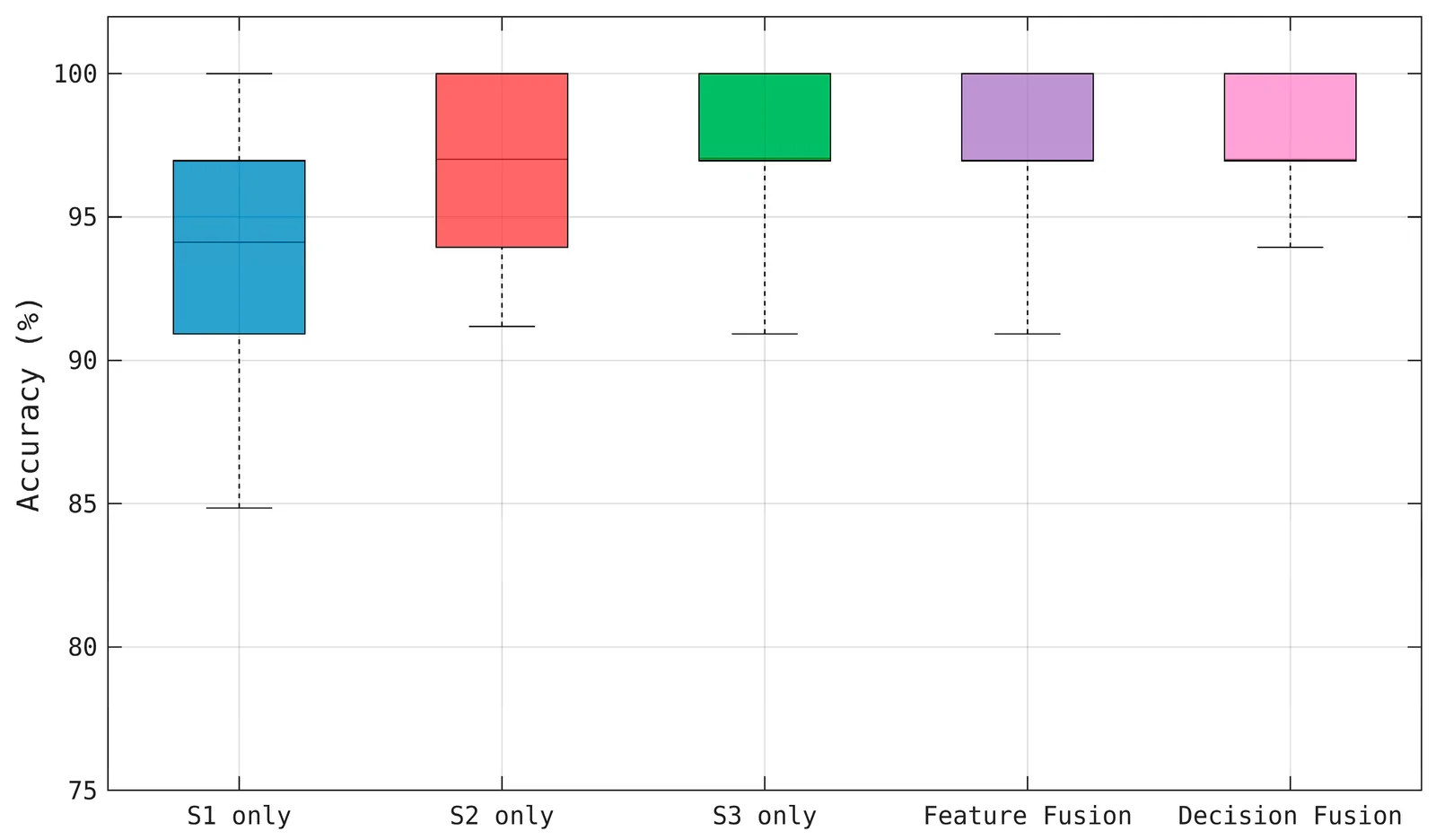
Classification accuracy across stratified 10-fold cross-validation.

The distributions of classification accuracy obtained from repeated holdout and stratified 10-fold cross-validation are shown in Figure 3 and Figure 4, respectively. The corresponding quantitative results, including the fusion models, are summarized in Table 4.

Both fusion strategies achieved higher mean accuracy than the S1 and S2 individual-channel models under both validation schemes. Feature-level fusion produced the highest mean accuracy, reaching 97.12 ± 2.19% under repeated holdout evaluation and 97.91 ± 3.18% under 10-fold cross-validation. Equal-vote decision fusion achieved 96.72 ± 2.22% and 97.60 ± 2.36%, respectively.

The mean differences between feature-level and decision-level fusion were 0.40 percentage points under repeated holdout and 0.31 percentage points under 10-fold cross-validation. Their mean performances were therefore numerically similar, although no direct statistical-equivalence test between the two fusion strategies was conducted. The improvement of feature fusion over the strongest individual channel, S3, was also modest. Exploratory statistical sensitivity analyses are presented in Section 6.4. Across the 30 repeated holdout runs, 7.50 ± 1.91 test events per run contained at least one individual-channel classification error. Equal-vote fusion returned the correct class for 5.33 ± 1.77 of these events, corresponding to a mean run-wise correction percentage of 72.04 ± 16.80%. This percentage is the mean of the 30 run-wise percentages, not the ratio of the two reported mean event counts (5.33/7.50 = 71.1%). Conversely, fusion was incorrect despite at least one correct individual-channel prediction for 1.30 ± 1.15 events per run. These descriptive results show that voting often resolves non-identical channel errors but is not a guaranteed error-correction mechanism.

### 6.2. Predictor-Count and Feature-Redundancy Sensitivity Results

**Figure 5:**
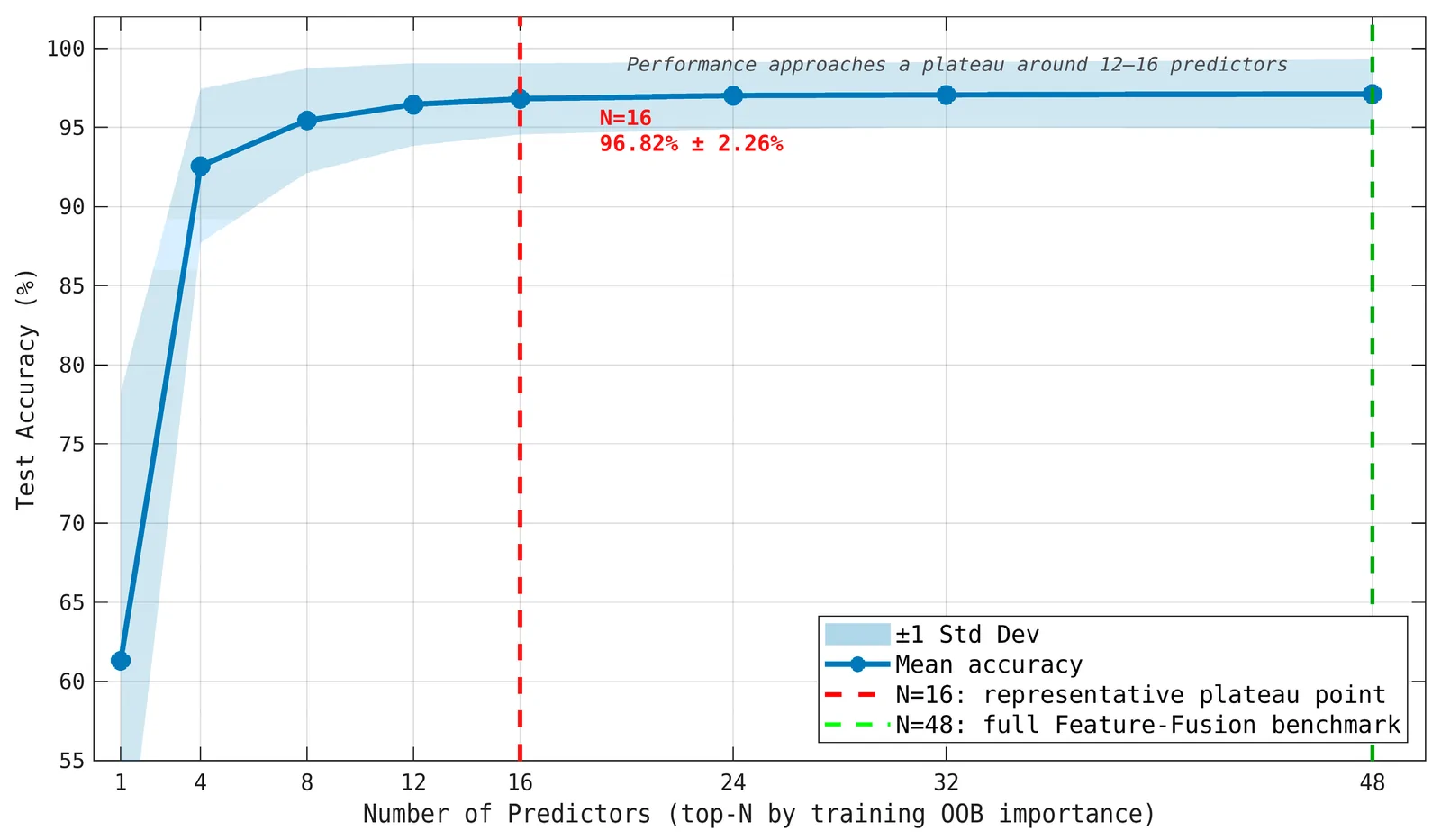
Predictor-count sensitivity of the feature fusion random forest. Rankings were derived from training-partition OOB permutation importance within each split; the 48-predictor point is the full-feature reference. Values are mean ± SD across 30 holdout runs.

To evaluate predictor-count sensitivity, the fused random forest model was retrained using progressively larger subsets of predictors ranked by training-partition OOB permutation importance. The resulting classification accuracy is shown in Figure 5.

Classification accuracy increased rapidly with predictor count, reaching 95.45 ± 3.31% with the top eight training-ranked predictors and then approaching a plateau: 96.46 ± 2.62% with 12 predictors, 96.82 ± 2.26% with 16, 97.02 ± 2.12% with 24, 97.07 ± 2.06% with 32, and 97.12 ± 2.19% for the full 48-feature reference. The 16-predictor mean was therefore 0.30 percentage points below the full reference, small relative to run-to-run variability. Because rankings were recomputed within every split, these results indicate predictor-count sensitivity rather than independent validation of one fixed compact feature set. Selection-frequency analysis further showed that eight predictors appeared among the top 16 in every training partition: S1 RMS, S1 Energy, S1 mean frequency, S1 0–100 kHz band power, S2 crest factor, S2 normalized active duration, S3 kurtosis, and S3 spectral bandwidth (Figure S1).

When the three energy predictors were removed, the 45-feature fusion model achieved 97.22 ± 2.22%, compared with 97.12 ± 2.19% for the full 48-feature model; the paired change was +0.10 ± 0.97 percentage points. The negligible difference indicates that the reported feature fusion accuracy did not depend materially on retaining both energy and RMS, although these correlated variables should still not be interpreted as independent physical evidence.

### 6.3. Sensor Frequency Analysis

**Figure 6:**
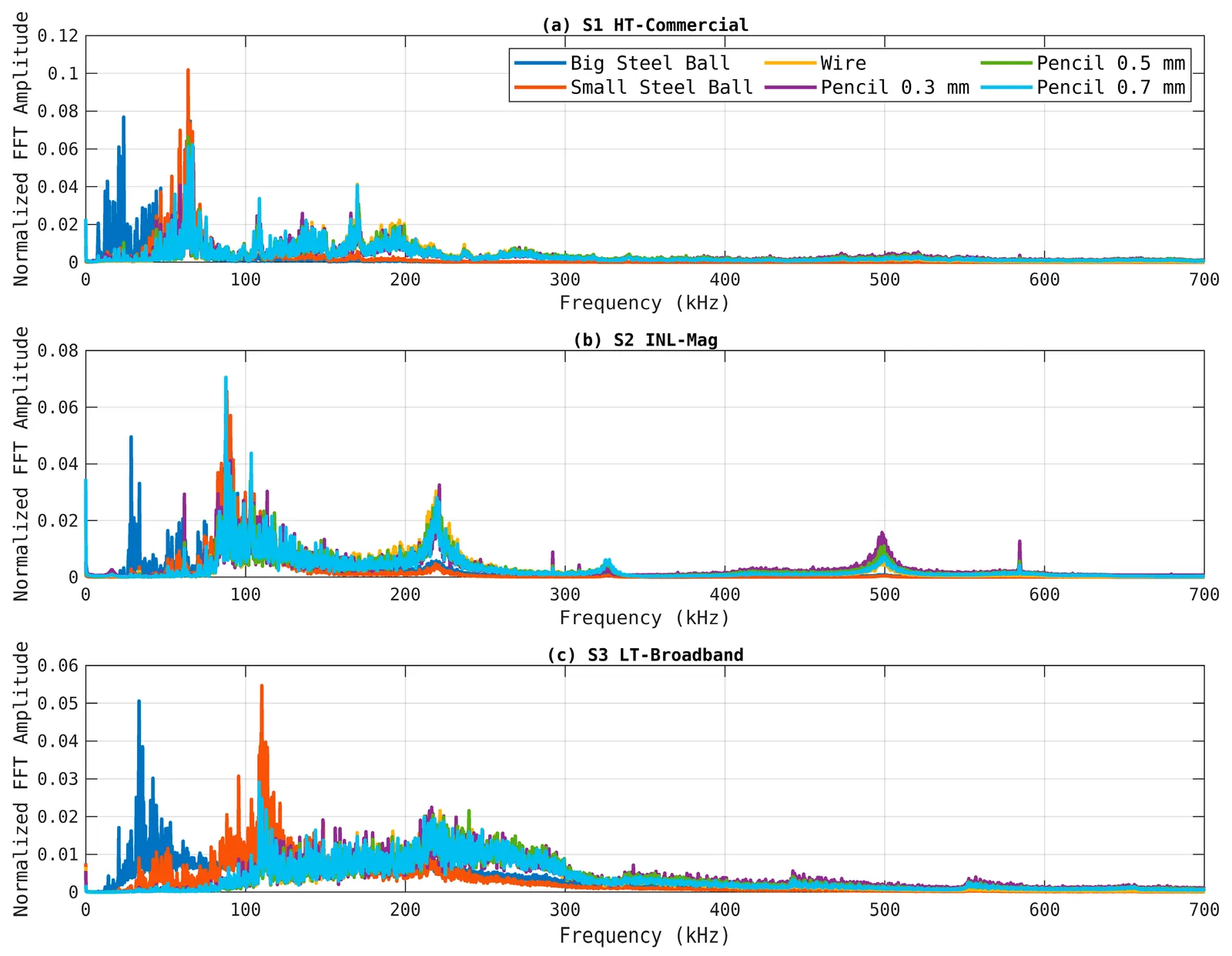
Mean L2-normalized FFT spectra for all six impact classes by sensor: (**a**) S1, (**b**) S2, and (**c**) S3. The common 30–700 kHz analog bandpass strongly attenuated content below approximately 30 kHz. No sensor-specific frequency masking was applied.

**Figure 7:**
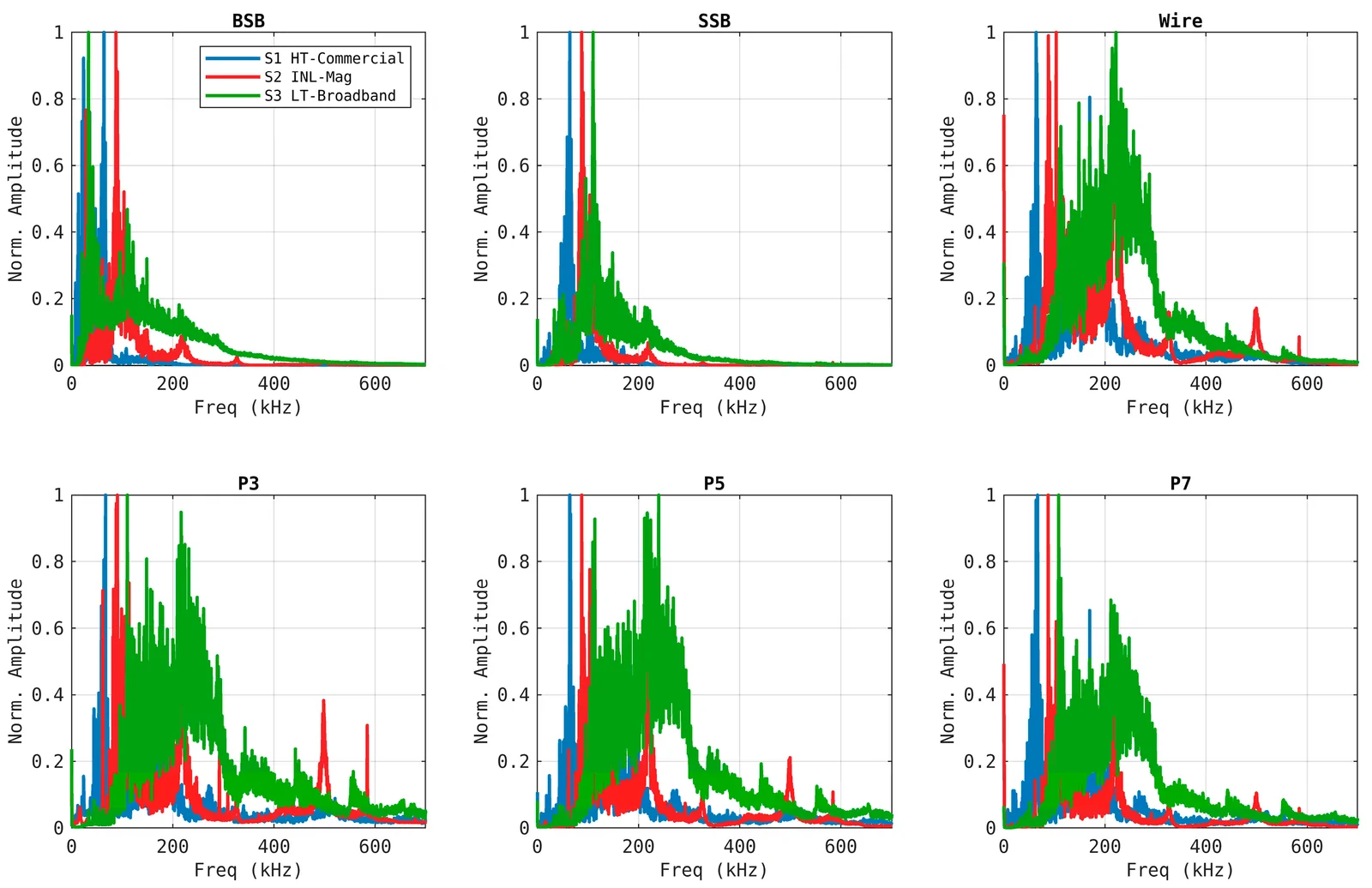
Peak-normalized spectral comparison of the three complete sensor channels for each impact class.

The three sensor channels exhibited distinct spectral responses despite recording the same impact events. Figure 6 and Figure 7 compare these responses using two complementary normalization approaches.

Figure 6 shows the mean spectrum for each impact class within each sensor channel after normalization by the spectrum’s L2 norm. This normalization emphasizes differences in spectral shape rather than absolute amplitude.

The magnetostrictive channel exhibited spectral shapes that differed from those of the commercial piezoelectric channels, particularly below approximately 300 kHz. Although isolated higher frequency peaks were also present, many of the broad class-dependent differences were concentrated in the lower portion of the retained frequency range.

These channel-specific responses provide a plausible basis for the numerical improvements obtained through fusion. However, sensor technology did not independently vary from spatial position, mounting, coupling, or propagation path. The observed spectral differences should therefore be interpreted as properties of the complete sensor channels under the tested configuration rather than effects attributable exclusively to transduction mechanism.

Because the spectra in Figure 6 and Figure 7 were normalized, these figures should not be used to compare absolute signal energy among channels. Absolute-amplitude information is represented separately by features such as RMS and waveform energy. The manufacturer-reported frequency ranges in Table 1 are nominal response or operating ranges, and the present spectra are not calibrated transfer-function measurements of the individual transducers. No sensor-specific frequency masking was applied.

### 6.4. Exploratory Statistical Sensitivity Results

Exploratory split-matched comparisons were performed using the 30 repeated holdout results. Because the repeated holdout partitions overlap and are not fully independent, these tests are secondary sensitivity analyses rather than confirmatory inference. Primary interpretation is based on the accuracy distributions and paired effect-size magnitudes. Raw and Holm-adjusted *p*-values are reported in Table 5 for transparency.

The largest split-matched effect sizes occurred for comparisons involving the high-temperature commercial channel S1, and the exploratory Holm-adjusted *p*-values for S2 versus S1, feature fusion versus S1, feature fusion versus S2, and decision fusion versus S1 were below 0.05. Because the repeated splits overlap, these *p*-values are not treated as independent confirmatory evidence; they are consistent with the accuracy distributions showing the clearest numerical differences relative to S1.

The mean difference between S2 and S3 was small, and the corresponding exploratory Holm-adjusted *p*-values exceeded 0.05. Feature fusion likewise showed only a modest mean improvement over S3. Accordingly, differences among the magnetostrictive channel, broadband commercial channel, and fusion models should be interpreted as comparatively modest under the tested configuration.

## 7. Discussion

### 7.1. Classification Performance of the INL Magnetostrictive Sensor

The principal finding is that, within the present single-specimen, single-campaign room-temperature dataset, the INL magnetostrictive sensor channel retained sufficient information for high-accuracy impact-source classification. As reported in Section 6.1, its mean accuracy was 2.98 percentage points higher than the high-temperature commercial channel and close to the broadband commercial channel under repeated holdout evaluation. These values compare complete sensor-channel responses under fixed, non-coincident mounting and therefore cannot be interpreted as intrinsic differences among transduction mechanisms. The exploratory statistical sensitivity analysis is secondary to the accuracy distributions and effect-size magnitudes.

These findings complement prior studies that demonstrated the harsh-environment potential of the INL sensor architecture, including high-temperature fuel-pin burst detection and irradiation testing of related magnetostrictive materials [10,11,12]. The present study did not independently test classification performance at elevated temperature or under irradiation, and room-temperature accuracy should not be used to infer retained accuracy under those conditions. The present results should therefore be viewed as a baseline classification study that motivates, but does not replace, harsh-environment validation.

To the authors’ knowledge, this work provides the first systematic machine-learning evaluation of the INL magnetostrictive AE sensor channel alongside commercial piezoelectric alternatives. The contribution is not a new machine-learning algorithm or a claim of universal sensor superiority; rather, it is evidence that a magnetostrictive sensor architecture developed for harsh nuclear environments can retain sufficient time- and frequency-domain information for automated AE source classification under the tested laboratory configuration.

### 7.2. Sensor-Channel Complementarity and Study Limitations

The three sensor channels provided partially complementary information under the tested configuration, as reflected by the small numerical improvement from fusion relative to the strongest individual channel. Each sensor occupied a fixed, non-coincident position, and sensor type was not independently varied from bandwidth, propagation path, mounting, or coupling; consequently, differences among the measured spectra, feature distributions, and classification results cannot be attributed solely to piezoelectric or magnetostrictive transduction. The measurements were also limited to one 304 stainless-steel structure, room temperature, controlled laboratory impact sources, one experimental campaign, and 55–57 events per class.

Because the source classes were acquired in class-specific blocks rather than randomized or interleaved order, temporal changes in coupling, instrumentation, or other experimental conditions may be associated with class identity; stratified partitioning does not remove this confound. Without repeated independently acquired blocks per class, the magnitude of a between-block effect is not identifiable separately from class effects in the present dataset. Accordingly, the reported validation establishes only within-campaign and within-specimen performance. Generalization requires new campaigns with randomized or interleaved repeated class blocks, multiple specimens and geometries, varied propagation distances and mounting conditions, position-controlled or co-located sensor comparisons, independent acquisition sessions, representative background noise, and elevated-temperature and radiation environments.

The within-block temporal screening, which used file row order as an unverified proxy for acquisition sequence, provides additional context rather than a remedy for this design limitation. Across the 288 class–sensor–feature combinations, the median |ρ| was 0.196 and the 90th percentile of |ρ| was 0.478; 29.2% of combinations had |ρ| ≥ 0.30 and 9.0% had |ρ| ≥ 0.50. The largest observed association was |ρ| = 0.683 for the P5/S1 peak-amplitude feature (Table S1; Figure S2 summarizes the corresponding class–sensor medians). Thus, some moderate-to-strong monotonic associations with the row-order proxy were present. Because class and block remain inseparable and this screening cannot detect constant between-block offsets, it does not rule out temporal confounding and reinforces the need for randomized or interleaved repeated blocks.

### 7.3. Feature Fusion Versus Decision Fusion

Feature-level fusion achieved the highest mean accuracy, while decision fusion trailed by only 0.40 percentage points under repeated holdout and 0.31 percentage points under 10-fold cross-validation; because no direct equivalence test was conducted, the two strategies should be described as numerically similar rather than statistically equivalent. Feature fusion can exploit cross-channel predictor relationships but requires all 48 inputs from a consistent feature pipeline. Decision fusion is more modular because each channel retains an independent model that can be evaluated, updated, or replaced separately, which may be useful in long-term monitoring systems, although sensor-dropout or missing-channel operation was not evaluated here. The small numerical gap between the two approaches suggests that the 16-feature representation already captures much of the discriminative information available from each channel before fusion. The aggregate 30-run diagnostic is consistent with this interpretation: among events with at least one individual-channel error, the mean run-wise equal-vote correction percentage was 72.04 ± 16.80%, but it also produced 1.30 ± 1.15 errors per run despite at least one correct individual prediction. Fusion should therefore be understood as a modest average robustness benefit, not a universal error-correction rule.

### 7.4. Feature Importance and Physical Interpretability

**Figure 8:**
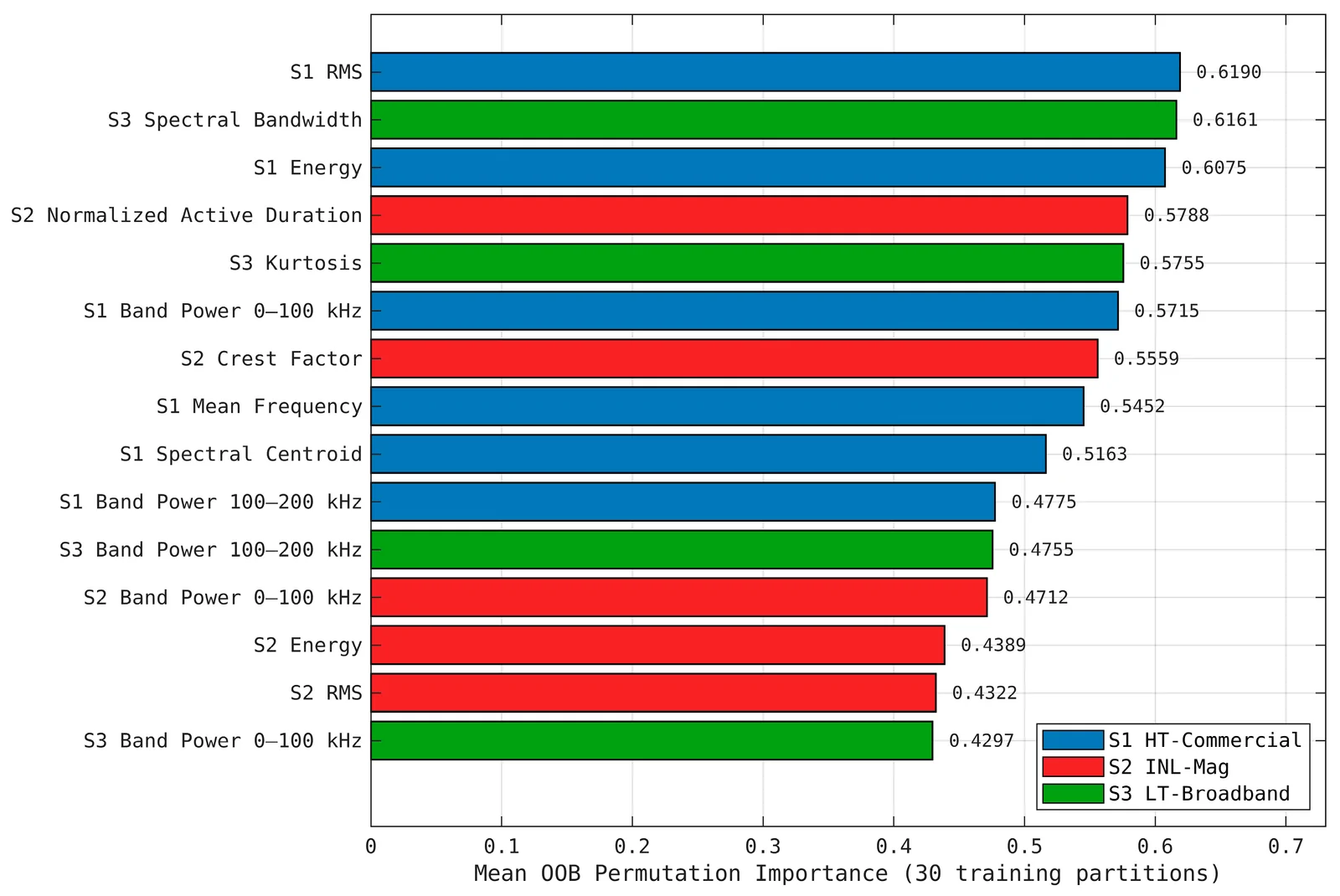
Top 15 predictors by mean training-only OOB permutation importance across 30 repeated-holdout training partitions.

Figure 8 shows the mean training-only OOB permutation-importance ranking of the 48-feature fused random forest model averaged across the 30 repeated-holdout training partitions.

Figure 8 shows that the fused model relied primarily on amplitude-related and low-frequency spectral predictors, including RMS, waveform energy, spectral bandwidth, low-frequency band power, and impulsiveness-related features. Among the 15 predictors shown, six originate from S1, five from S2, and four from S3. The three highest mean importance values were S1 RMS (0.6190), S3 spectral bandwidth (0.6161), and S1 Energy (0.6075). Energy and RMS should not be interpreted as independent evidence because they are deterministically related for equal-length records; more generally, OOB permutation importance can redistribute importance among correlated predictors. The ranking therefore characterizes how the fitted models used the available variables under this campaign and does not establish an independent physical or causal contribution for any individual feature.

Ranked predictors from all three channels indicate that each contributed information under the tested configuration, consistent with the spectral differences reported in Section 6.3; however, because sensor type was not separated from position, mounting, coupling, and propagation path, the ranking does not isolate transduction mechanism as the cause. The predictor-selection stability analysis showed that eight predictors appeared in the top 16 in every training partition (Figure S1), but the remaining identities varied by split and the set includes correlated variables. The energy-removal sensitivity analysis produced essentially unchanged accuracy (97.22 ± 2.22% versus 97.12 ± 2.19%), supporting the conclusion that classification performance did not depend materially on retaining both members of the RMS/Energy pair. A fixed reduced model should nevertheless use a deliberately nonredundant subset and be validated on independent data.

### 7.5. Offline Implementation Check

An additional software implementation check was performed in LabVIEW 2026 Q1 (National Instruments, Austin, TX, USA) using a MATLAB Script Node and prerecorded signals. The implementation reproduced the MATLAB feature-extraction, normalization, and random forest inference sequence for the full feature pipeline. This check was limited to offline numerical reproducibility; continuous acquisition, inference latency, sustained throughput, processor loading, and missed-trigger behavior were not evaluated. Accordingly, the LabVIEW check is not treated as a principal contribution or as evidence of real-time deployment.

## 8. Conclusions

This study evaluated whether an INL magnetostrictive AE sensor architecture developed for harsh-environment applications retains sufficient information for machine-learning-based impact-source classification under a controlled room-temperature laboratory configuration. Using 334 synchronized acquisitions from six impact classes, the magnetostrictive channel achieved high within-campaign classification accuracy and performance numerically comparable to the commercial piezoelectric channels. Because the three sensors occupied fixed, non-coincident positions, these comparisons represent complete sensor-channel responses and do not isolate transduction mechanism. Prior studies establish the harsh-environment potential of the sensor architecture [10,11,12], but the present study does not validate classification performance at elevated temperature or under irradiation.

Feature-level fusion achieved the highest mean accuracy, reaching 97.12 ± 2.19% under repeated holdout evaluation and 97.91 ± 3.18% under stratified 10-fold cross-validation. Equal-vote decision fusion produced numerically similar mean performance. Predictor-count sensitivity analysis showed 96.82 ± 2.26% mean accuracy with 16 training-ranked predictors versus 97.12 ± 2.19% for all 48 predictors within the same repeated-holdout framework; this does not define or independently validate a fixed compact feature set. Removing the three energy predictors produced 97.22 ± 2.22%, indicating that the reported fusion performance was not materially dependent on retaining both RMS and energy. The observed fusion benefit reflects complementary information among the complete sensor channels under the tested configuration and cannot be attributed solely to transduction mechanism.

The present event-level accuracies should be interpreted as within-campaign and within-specimen results because class-specific acquisition blocks were not randomized or interleaved. They may be optimistic and do not establish block-independent or operational generalization. Future validation should therefore include repeated randomized or interleaved class blocks, independent acquisition campaigns, multiple specimens and structural geometries, varied mounting and propagation conditions, position-controlled comparisons using swapped, rotated, or co-located sensor placements, representative background noise, elevated-temperature and radiation testing, live-acquisition performance measurements, and model-interpretation studies. The current results provide a laboratory baseline for that validation rather than evidence of generalization to reactor operating conditions. A descriptive screen using file row order as an unverified proxy for within-block acquisition sequence found some moderate-to-strong feature associations and therefore reinforces, rather than eliminates, the acquisition-block limitation.

## Figures and Tables

**Table 1:** Sensor specifications used in this study [11,12,29,30].

Sensor	Type	Designation	Key Characteristics
Sensor 1	High-temperature commercial piezoelectric (MISTRAS D9215)	S1 (HT-Commercial)	Differential piezoelectric response; 50–650 kHz operating range, rated operating temperature −200 to 540 °C; radiation-resistant
Sensor 2	INL magnetostrictive AE sensor	S2 (INL-Mag)	Inverse magnetostrictive (Villari) effect; Fe-Co alloy; Curie temperature approximately 950 °C; radiation-tolerant; AE detection demonstrated up to 650 °C
Sensor 3	Low-temperature broadband commercial piezoelectric (MISTRAS WD)	S3 (LT-Broadband)	Wideband response; 100–900 kHz good-response range and 125–1000 kHz operating range; reference commercial baseline; rated operating temperature −65 to 177 °C

**Table 2:** Acquisition parameters.

Parameter	Value
Sampling frequency	20 MHz
Samples per acquisition	1,100,000 per channel
Recording window	55 ms
Number of sensors	3, acquired simultaneously
Number of impact classes	6
Usable acquisitions per class	55–57
Total dataset size	334 usable acquisitions
Trigger mode	Threshold crossing (any channel)
Preamplifier	MISTRAS 2/4/6, 60 dB gain, 30–700 kHz bandpass filter
Oscilloscope/digitizer	PicoScope 4824A

**Table 3:** Spectral band-power feature definitions.

Feature	Sub-Band (kHz)	Physical Interpretation
$f_{12}$	0–100	Lowest retained band; content below approximately 30 kHz attenuated by common analog high-pass
$f_{13}$	100–200	Lower analyzed AE band
$f_{14}$	200–300	Intermediate analyzed AE band
$f_{15}$	300–500	Higher frequency AE content
$f_{16}$	500–700	Highest retained frequency band

**Table 4:** Classification accuracy of the individual sensor-channel and fusion models.

Model	Features	30-Run Holdout Mean ± SD	10-Fold CV Mean ± SD
S1 (HT-Commercial)	16	92.37% ± 2.94%	93.41% ± 4.23%
S2 (INL Magnetostrictive)	16	95.35% ± 2.45%	97.01% ± 3.14%
S3 (LT-Broadband)	16	96.31% ± 1.98%	97.01% ± 2.84%
Feature Fusion (FF-RF)	48	97.12% ± 2.19%	97.91% ± 3.18%
Decision Fusion—Equal Vote	3 × 16	96.72% ± 2.22%	97.60% ± 2.36%

**Table 5:** Exploratory paired comparisons across 30 repeated holdout runs.

Comparison	*t*-Test *p*	Wilcoxon *p*	Cohen’s $d_{z}$	Effect Size
S2 (INL-Mag) vs. S1 (HT-Commercial)	<0.001 (Holm < 0.001)	<0.001 (Holm < 0.001)	0.898	Large
S2 (INL-Mag) vs. S3 (LT-Broadband)	0.037 (Holm 0.074)	0.046 (Holm 0.093)	−0.399	Small
Feature Fusion vs. S1	<0.001 (Holm < 0.001)	<0.001 (Holm < 0.001)	1.659	Large
Feature Fusion vs. S2	<0.001 (Holm < 0.001)	<0.001 (Holm < 0.001)	0.887	Large
Feature Fusion vs. S3	0.058 (Holm 0.074)	0.082 (Holm 0.093)	0.361	Small
Decision Fusion vs. S1	<0.001 (Holm < 0.001)	<0.001 (Holm < 0.001)	1.651	Large

## Data Availability

The data and MATLAB R2024b analysis code supporting the findings of this study are available from the corresponding author upon reasonable request, subject to Idaho National Laboratory data-sharing, software-release, and other applicable institutional restrictions.

## Supplementary Materials

The following supporting information can be downloaded at: https://www.mdpi.com/article/10.3390/s26185693/s1, Table S1: Summary of additional sensitivity and diagnostic analyses performed using the existing dataset; Figure S1: Selection frequency for the 15 predictors most frequently ranked among the top 16 across the 30 repeated-holdout training partitions; Figure S2: The 18 class–sensor medians from the within-block row-order proxy screening.
