# Supplementary material for: Machine-Learning Evaluation of a Magnetostrictive Acoustic-Emission Sensor Developed for Nuclear Structural Health Monitoring
Source: Sensors (Basel). 2026 Sep 8;26(18):5693. doi: 10.3390/s26185693 (PMC13611483; doi:10.3390/s26185693)
Supplement: Supplementary file 1 [file sensors-26-05693-s001.zip › sensors-4500860-supplementary.pdf]

Supplementary Material

Machine-Learning Evaluation of a Magnetostrictive Acoustic-Emission Sensor Developed for Nuclear Structural Health Monitoring

Bibo Zhong, Chaitee Milind Godbole, Vivek Agarwal, and Joshua E. Daw

Existing-Data Sensitivity Diagnostics

The supplementary figures below provide descriptive diagnostics added in response to peer review. They use the existing synchronized dataset and do not remove the experimental-design limitations associated with fixed, non-coincident sensor positions or class-specific acquisition blocks.

Abbreviations and class labels: FF-RF, feature-fusion random forest; RMS, root mean square; S1 (HT-Commercial), high-temperature commercial piezoelectric sensor channel; S2 (INL-Mag), INL magnetostrictive sensor channel; S3 (LT-Broadband), low-temperature broadband commercial piezoelectric sensor channel; BSB, 10 mm big steel ball; SSB, 3 mm small steel ball; Wire, 0.6 mm tungsten-wire contact source; and P3, P5, and P7, 0.3, 0.5, and 0.7 mm pencil-lead-break sources, respectively.

Table S1. Summary of additional sensitivity and diagnostic analyses using the existing dataset.

Diagnostic	Result	Interpretation
30-run equal-vote fusion behavior	Across the 30 runs, 7.50 ± 1.91 test events/run had ≥1 individual-channel error; fusion correctly classified 5.33 ± 1.77 of these events/run. The mean of the 30 run-wise correction percentages was 72.04 ± 16.80%. Fusion also produced 1.30 ± 1.15 errors/run despite ≥1 correct individual prediction.	Fusion often resolves non-identical channel errors, but is not a universal error-correction mechanism.
Energy-removal sensitivity (RMS retained)	Full 48-feature FF-RF: 97.12 ± 2.19%; Energy removed (45 features): 97.22 ± 2.22%; paired change (Energy-removed minus full model, matched by run): +0.10 ± 0.97 percentage points.	Retaining both deterministic RMS/Energy descriptors did not materially affect feature-fusion accuracy.
Within-block row-order proxy screening	Across 288 correlations (6 classes × 3 channels × 16 features), median $\lvert\rho\rvert = 0.196$; 90th percentile = 0.478; 29.2% with $\lvert\rho\rvert \geq 0.30$; 9.0% with $\lvert\rho\rvert \geq 0.50$; maximum feature-level $\lvert\rho\rvert = 0.683$ for P5/S1 peak amplitude.	Some associations with the row-order proxy are present. This screening cannot estimate between-block offsets, verify chronology, or remove class/block confounding.

Statistical notation: Values reported with ± are mean ± standard deviation (SD) across 30 repeated-holdout runs. The paired change was calculated within each matched run as the 45-feature Energy-removed model accuracy minus the full 48-feature model accuracy. The fusion correction percentage was calculated within each run and then averaged; therefore, 72.04% is not the ratio of the reported mean counts, 5.33/7.50. Within-file row order was used as an unverified proxy for acquisition sequence.

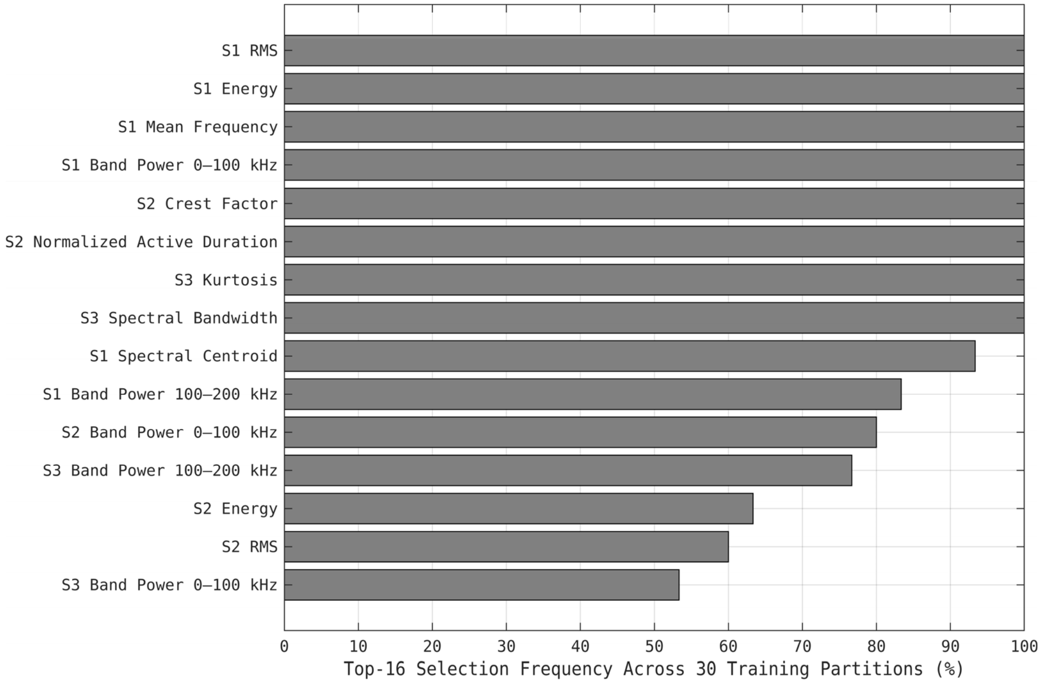

Figure S1. Selection frequency of the 15 most frequently selected predictors across 30 repeated-holdout training partitions. Within each partition, predictors were ranked using the training data only, and selection frequency was the percentage of partitions in which a predictor appeared among the top 16. Eight predictors appeared among the top 16 in all 30 partitions.

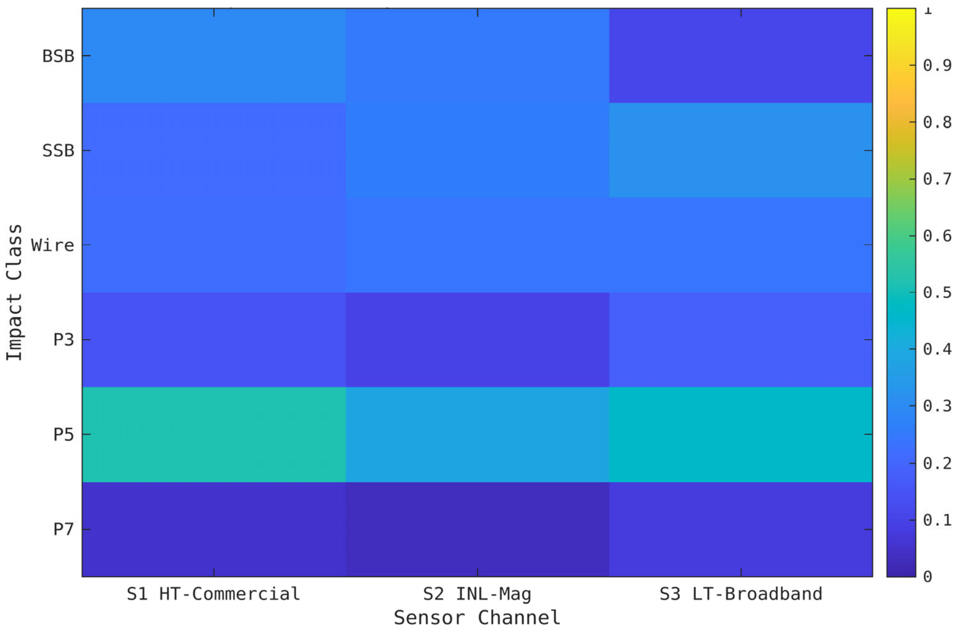

Figure S2. Median absolute Spearman rank-correlation coefficients ($|\rho|$) with the within-file row-order proxy, summarized by class and sensor channel. Each of the 18 class–sensor cells shows the median of 16 absolute Spearman rank-correlation coefficients, one per extracted feature, between the within-file row-order proxy and feature value. Thus, the heat map summarizes 288 correlations (6 classes × 3 sensor channels × 16 features). The feature-level maximum, $|\rho| = 0.683$ for P5/S1 peak amplitude, is reported in Table S1; it is not displayed as a separate cell because the heat map shows class–sensor medians.
